# Sleep and Mental Health Issues in Current and Former Athletes: A Mini Review

**DOI:** 10.3389/fpsyg.2022.868614

**Published:** 2022-04-07

**Authors:** Ashley Montero, David Stevens, Robert Adams, Murray Drummond

**Affiliations:** ^1^College of Education, Psychology and Social Work, Flinders University, Bedford Park, SA, Australia; ^2^Sport, Health, Activity, Performance and Exercise (SHAPE) Research Centre, Flinders University, Bedford Park, SA, Australia; ^3^Adelaide Institute for Sleep Health, Flinders University, Bedford Park, SA, Australia

**Keywords:** help-seeking, stigma, athletic retirement, anxiety, depression, suicide, insomnia, obstructive sleep apnoea (OSA)

## Abstract

Sleep and mental health are important aspects of human health that work concurrently. However, sleep and mental health disorders are often overlooked and undiagnosed in sport due to the negative stigma associated with them. Evidence suggests that athletes are disproportionately affected by mental health issues and sleep problems. Internal and external pressures contribute to psychological distress. Variable competition times, travel and stress are detrimental to sleep quality. Retirement from sport can deteriorate sleep and psychological wellbeing, particularly for those who retired involuntarily and identify strongly with their athletic role. When untreated, these issues can manifest into a range of clinical disorders. This is concerning, not only for compromised athletic performance, but for general health and wellbeing beyond sport. Previous research has focussed on sleep and health independently among currently competing, or former, athletes. To date, no research has comprehensively assessed and compared sleep complaints and mental health issues between these two cohorts. Moreover, research has failed to obtain data across a variety of different competition levels, sports, and genders, leaving the current scope of the literature narrow. Comorbid conditions (e.g., concussion history, obesity), ex-college athletes, and mental health has been the focus of existing literature post-retirement. Future research would benefit from employing both quantitative and qualitative methodologies to comprehensively assess the prevalence and severity of sleep and mental health disorders across current and retired athletes. Research outcomes would inform education strategies, safeguarding athletes from these issues by reducing negative stigmas associated with help-seeking in sport and ultimately increase self-guided treatment.

## Highlights

- Current and retired athletes appear more predisposed to both sleep and mental health disorders.
- Involuntary retirement, high athletic identity, and social withdrawal after leaving sport can trigger or exacerbate psychological distress and sleep problems.
- Athletes fail to report sleep and mental health issues due to fear of negative evaluation, the perceived repercussions of seeking help, lack of mental health literacy, and limited knowledge of where or how to get support.
- Previous research has failed to comprehensively compare sleep and mental health symptomology and prevalence between current and retired athletes.
- Future research utilising consistent and validated tools is needed to accurately measure sleep and mental health issues, allowing early identification of potential disorders before they manifest.

## Introduction

The overall sleep health in the general population across the globe is poor (Adams et al., 2016; Chattu et al., 2019). Poor sleep behaviours and clinical sleep disorders, such as insomnia and sleep disordered breathing, are associated with deleterious health outcomes, including hypertension, diabetes, stroke, psychological impairment, and psychiatric disorders (Kendzerska et al., 2013; Khurshid, 2018). For athletes, poor sleep has the added burden of diminished athletic performance, impaired recovery, and increased injury risk (Malhotra, 2017; Lastella et al., 2018; Bolin, 2019; Vitale et al., 2019; Huang and Ihm, 2021; Costa et al., 2022). Like the general population, a bi-directional relationship between sleep and mental health has been observed amongst athletes (Asplund and Chang, 2020). Sleep dysfunction is positively correlated with depression, anxiety, and worry in sport (Benjamin et al., 2020); while conversely, psychiatric conditions can directly disturb sleep (Asplund and Chang, 2020). Depression, for example, has shown to alter sleep architecture at both a macro level (i.e., stage duration) and micro level (i.e., spindle activity) (Wichniak et al., 2013). Importantly for athletes, the cyclical nature of poor sleep and mental ill-health can significantly hinder sporting performance and manifest into a more serious disorder, becoming a burden to general health and wellbeing (Asplund and Chang, 2020).

While there has been a considerable amount of research separately examining sleep and mental health problems in current athletes (Gulliver et al., 2014; Cheek et al., 2015; Kong and Harris, 2015; Rao and Hong, 2016; Swinbourne et al., 2016; Kilic et al., 2017; Malhotra, 2017; Thornton et al., 2017; Tuomilehto et al., 2017; Foskett and Longstaff, 2018; Castaldelli-Maia et al., 2019; Asplund and Chang, 2020; Caia et al., 2020; Timpka et al., 2021; Costa et al., 2022), almost none has examined the two areas together. Furthermore, although there is some research on mental health problems in retired athletes (Kerr et al., 2014a; Gouttebarge et al., 2016; Kilic et al., 2017; Fernandes et al., 2019; Mannes et al., 2019; Esopenko et al., 2020), little is known about mental health or sleep problems outside of comorbid conditions (i.e., concussion history, obesity) in ex-contact sport athletes (Hyman et al., 2012; Kerr et al., 2012, 2014b; Didehbani et al., 2013; Hart et al., 2013; Strain et al., 2013; Iverson, 2014; Churchill et al., 2018; Hutchison et al., 2018; Hind et al., 2021; Schaffert et al., 2021). This is despite current and former athletes being susceptible to both sleep and mental health disorders (Gouttebarge et al., 2019). Therefore, the aim of this paper is to present the current literature on these disorders during and after an athletic career. In turn, gaps in the literature can be addressed, to which the subsequent need for future research can be identified.

## Research Strategy

This review was conducted using an initial systematic approach and subsequently broadened to ascertain the scope of the current literature on sleep and mental health in current and retired athletes. The following key terms were initially searched in several databases including SPORTDiscus, PubMed, Scopus, Flinders University online library, and Google Scholar: sleep OR insomnia OR apnoea OR apnoea OR OSA OR “periodic leg movement disorder”; “mental health” OR depression OR anxiety OR “quality of life” OR suicide OR wellbeing; AND sport OR athlete; AND retired OR former OR current. Articles met the following criteria: published after 2011; research papers; published in English language; relevant to the research objectives. Further investigation into these topics allowed insight into additional research areas, the extent to which they have been studied, and theoretical framework explaining why these issues occur.

### Sleep and Mental Health in Current Athletes

Athletes experience less sleep than they require, and the quality of their sleep is generally poor (Randell et al., 2021; Sargent et al., 2021; Walsh et al., 2021). Training schedules, competition timing, and travel contribute to their variable and poor sleep (Juliff et al., 2014; Sargent et al., 2014, 2022; Horváth et al., 2016; Nedelec et al., 2018). High stress, worry and anxiety from competitive sport can increase sleep latency, further impairing sleep quality (Nedelec et al., 2018). The demands of sport that impair sleep can manifest into a clinical sleep disorder. As a result, high prevalence of sleep disorders is observed in athletic populations (Silva et al., 2019).

Insomnia and obstructive sleep apnoea (OSA) are common sleep disorders. Insomnia affects approximately 15% of the adult population (Pallesen et al., 2013; Reynolds et al., 2019), whilst OSA affects approximately one billion people worldwide, with population prevalence exceeding 50% in some cohort studies (e.g., China, United States, and Brazil) (Benjafield et al., 2019). Research suggests that athletes (male athletes in particular), however, are more predisposed to sleep disorders. Insomnia symptoms are reported between 27 and 37% of athletes (Khalladi et al., 2019; Silva et al., 2019; Ballesio et al., 2021), whilst sleep maintenance insomnia has been reported in 77% athletes (Thornton et al., 2017). The psychological demands of sport, performance anxiety and overthinking delaying sleep behaviours can play a role in the development of these symptoms (Asplund and Chang, 2020). Within athletes, up to 45% report, or have shown to suffer from, OSA (Thornton et al., 2017; Tuomilehto et al., 2017; Caia et al., 2020); however, previous OSA research in sport has focussed on collision sports (e.g., American football, rugby). Players of these sports often have large body mass indices (>28 kg/m^2^) and neck circumference (>40 cm) (Swinbourne et al., 2016), which despite being advantageous to participate in collision sports, do predispose these athletes to not only OSA, but other health issues later in life (Swinbourne et al., 2016). Importantly, the prevalence of sleep disorders within athletic populations is thought to be under reported, with the field still under-researched and evidently, many athletes fail to report sleep problems.

Elite athletes are confronted with 640 distinct stressors that can induce mental health issues (Arnold and Fletcher, 2012). Athletes are subject to scrutiny from mainstream and social media, coaches, and themselves, which can take a toll on psychological wellbeing (Arnold and Fletcher, 2012; Siekanska et al., 2013). Excessive criticism from coaches has also been identified to significantly hinder athletic development (Siekanska et al., 2013). Internal pressures that athletes place on themselves and the external criticism received from others can be psychologically damaging and enduring. Consequently, these high psychological demands can culminate into a mental health disorder. As a result of this, athletes are suggested to be more predisposed to psychiatric disorders than the general population (Gulliver et al., 2014; Foskett and Longstaff, 2018; Castaldelli-Maia et al., 2019; Gouttebarge et al., 2019; Purcell et al., 2019).

Depression affects 4.4% of the global population, while anxiety-related conditions affect 3.6% of the population (World Health Organization, 2017). Athletes appear more susceptible to these disorders. A survey of Australian athletes showed a high prevalence of depression (27.2%), eating disorders (22.8%), social anxiety (14.7%), and generalised anxiety disorder (7.1%) (Gulliver et al., 2014)—significantly greater than depression (10.4%) and anxiety (13.1%) rates in the Australian population (Australian Bureau of Statistics, 2018). Depression and anxiety prevalence within some cohorts of athletes have shown to be as high as 47.8% (Foskett and Longstaff, 2018). Mental health issues are often co-morbid with other disorders. Suicide ideation is predicted by sleep problems, insomnia, depression, and anxiety (Khader et al., 2020; Wiebenga et al., 2021). Worryingly, the association with sleep problems and suicide ideation is stronger amongst college athletes than non-athletes (Wiebenga et al., 2021). Thus, it is unsurprising that 15.6% of collegiate athletes report suicide ideation, with some unfortunately, attempting and committing suicide (Rao and Hong, 2016; Timpka et al., 2021; Wiebenga et al., 2021). The bi-directional relationship between mental health and sleep can explain the high rates of sleep and mental health disorders observed amongst athletes. Moreover, if help is not sought, a seemingly never-ending cycle where mental health issues exacerbate sleep disorders (and vice versa) exists.

Clinical eating disorders are complex psychiatric conditions where onset is often attributed to a multitude of factors, including sleep. Insomnia increases the risk of eating disorders, while eating disorders further disrupted sleep (Allison et al., 2016). Eating disorders in sport affect more females (up to 45%) than males (up to 19%) (Bratland-Sanda and Sundgot-Borgen, 2013); often, coaches play a role in their development. Further, over 60% of elite athletes from both leanness focussed (e.g., dance, gymnastics) and non-leanness focussed sports (e.g., ball sports) reported pressures from coaches regarding their body shape (Bratland-Sanda and Sundgot-Borgen, 2013). Consequently, overt weight-related coaching pressures increase reports of poor mental health, fatigue, depression, and insufficient sleep (Cheek et al., 2015). Furthermore, the onset of psychological and sleep disorders amongst athletes can often be attributed to external sources. Unfortunately, many of these problems persist into retirement.

### Sleep and Mental Health in Retired Athletes

Athletic retirement often happens either: voluntarily (e.g., family, or social commitments, new career direction), or involuntarily (e.g., serious injury, age, dropped from the team due to subpar performance) (Kaul, 2017). Further, the outcome of this transition can be classified as either: successful (i.e., transition demands are met), or crisis (i.e., ineffective coping) (Stambulova and Samuel, 2019; Cosh et al., 2021). Voluntarily retiring from sport can be a positive change for some. Moreover, exiting sport on an athlete’s own terms can be a form of relief and an escape from the high physical and psychological demands of sport (Jones and Denison, 2017). For many, athletic retirement can be a difficult time to navigate—often resulting in crisis (Eggleston et al., 2020). Difficulty upon retirement from sport is correlated with high athletic identity (Giannone et al., 2017)—the degree to which an athlete identifies with their athletic role (Brewer and Petitpas, 2017). Many athletes put extensive time and energy into sport, so much so that they may not engage in alternatives outside of sport, hindering the establishment of self-identity (Hatamleh, 2013). Men tend to identify more strongly with an athletic role than women (Sturm et al., 2011); therefore, male athletes may experience distress during retirement more often than female athletes. For full-time professional athletes and those forced to retire, this transition is often more challenging (Eggleston et al., 2020). Unsuccessful, crisis transitions out of sport can explain why retired athletes are more susceptible to mental health issues than the general population (van Ramele et al., 2017; Ivanović and Maěak, 2020).

Involuntary retirement often leaves an athlete emotionally unprepared for life beyond sport (Gervis et al., 2019; Esopenko et al., 2020). With career-ending injuries, post-retirement planning is often not yet considered due to the expectation that the athlete’s career would not end so abruptly (Kuettel et al., 2017; Stambulova and Samuel, 2019). For full-time professional athletes, career termination means that their primary source of income is lost; therefore, they are not only mentally, but financially unprepared (Eggleston et al., 2020). Further, the breakdown of the social networks and sudden loss of identity formed throughout a sporting career can act as a catalyst for psychological distress following retirement (Brewer and Petitpas, 2017), thwarting the coping mechanisms necessary for adaptation. Importantly, athletic retirement can elicit psychological and emotional issues, transpiring into sleep problems (Fernandes et al., 2019), disordered eating (Buckley et al., 2019), substance abuse (Mannes et al., 2019), anxiety (Hart et al., 2013), depression (Giannone et al., 2017), and ultimately suicide (Malcolm and Scott, 2012). Mortality rates of retired athletes have reported to be 2–4 times higher than the general population (Lindqvist et al., 2014). While we understand why sleep and mental health issues can transpire following retirement from sport, there is a lack of quantifiable research in the field to indicate the prevalence and severity of these issues.

Psychologically challenging athletic retirement can subsequently trigger sleep problems. Despite limited research in retired athletes, sleep disturbances appear to be prevalent. Retired National Football League (NFL) players were 2.5 times more likely to have sleep apnoea than a matched community cohort (Luyster et al., 2017). Another study showed 42% of ex-NFL players suffered comorbid hypertension and OSA (Hyman et al., 2012). Insomnia is under researched in retired athletes, although it goes together with the psychological distress and worry that involuntary, crisis exits from sport can generate. A recent meta-analysis identified 20.9% of former elite athletes had sleep disturbances (Gouttebarge et al., 2019)—an indication of insomnia symptomology. Insomnia is often comorbid with mental health issues, which can be severe and frequent following retirement. Anxiety and depression symptoms have shown to be worse and more common post-retirement, with rates being as high as 39% in former athletes (Kerr et al., 2012, 2014a; Gouttebarge et al., 2015, 2016; Kilic et al., 2017; Willer et al., 2018). Further, despite 75% of retired gymnasts and swimmers surveyed by Papathomas et al. (2018) being within a healthy weight range, 55% were dissatisfied with their body, and 60% were actively trying to lose weight. However, perceived quality of life in ex-athletes is suggested to be comparable to the general population (Filbay et al., 2019). Post-retirement research is typically centred around mental health and understanding why these issues transpire, not necessarily measuring them. Sleep disorders are under researched in this context, and are rarely measured simultaneously with mental health, despite the strong, bi-directional relationship between them (Asplund and Chang, 2020).

### Importance of Measuring Sleep

Sleep behaviours can indicate mental health problems. Sleep architecture can be analysed in two ways: at a macro level, where values such as overall sleep stage composition and sleep efficiency are calculated, and at a micro level, which provides precise details on actual waveforms that comprise sleep (Djonlagic et al., 2021). Depression has shown to impair both sleep macro and micro architecture, altering the structure, timing, and composition of sleep periods, resulting and shorter and poorer quality sleep (Wichniak et al., 2013; Medina et al., 2014; Pandi-Perumal et al., 2020). Since athletes are disproportionately affected by mental health disorders (Gulliver et al., 2014; Foskett and Longstaff, 2018; Castaldelli-Maia et al., 2019; Gouttebarge et al., 2019; Purcell et al., 2019), their sleep is inevitably suffering as a result. Yet, sleep microarchitecture has not been assessed in sport. To do this, polysomnography (the gold-standard of sleep measurement) is required but it is impractical for athletes (Sargent et al., 2016; Claudino et al., 2019). Polysomnography is costly and uncomfortable, which can subsequently change the sleep pattern; hence why polysomnography is not preferred for measuring athletes’ sleep longitudinally (Sargent et al., 2016; Claudino et al., 2019). Macro sleep architecture research within sport has been made easier by advancements in wearable device technologies (Sargent et al., 2016), but has failed to examine how sleep periods are influenced by mental health. This is important as poor sleep impairs performance, hinder recovery, precipitate injury, and exacerbate mental health issues (Malhotra, 2017; Lastella et al., 2018; Bolin, 2019; Vitale et al., 2019; Benjamin et al., 2020; Huang and Ihm, 2021; Costa et al., 2022). Furthermore, athletes will be less likely to seek necessary treatment if they are unaware that an issue is present.

### Barriers to Seeking Help

Stigma and lack of mental health literacy are significant barriers to athletes receiving help for mental health issues (Gulliver et al., 2014; Castaldelli-Maia et al., 2019). This stigma is more apparent in male athletes, exacerbated by hypermasculinity (Wahto et al., 2016). Society tends to place emphasis on male masculinity in sport, whereby sport is considered an important masculinising process where young boys are taught to accept values such as competitiveness, toughness, and a winning at all cost mentality (Agnew and Drummond, 2015). Meanwhile, a lack of acceptance of women as athletes is a commonly reported influencer of mental health in elite sport (Castaldelli-Maia et al., 2019). Seeking help can be considered a “weakness” (López and Levy, 2013), which does not align with characteristics that historically define masculinity. Therefore, athletes avoid seeking help for fear of being negatively evaluated by coaching staff and teammates (López and Levy, 2013). For example, one in five elite rugby code athletes reported that they would not seek help if they had a problem or were upset (Hind et al., 2021). Athletes consider it more acceptable to see a psychologist for performance or goal-setting reasons than seeking help for depression (Gulliver et al., 2014). These issues continue to manifest when untreated, resulting in many athletes engaging in unhealthy coping mechanisms, such as substance abuse and overtraining (Wolanin et al., 2015); substance abuse has shown to persist, and even worsen post-retirement (Kerr et al., 2014a; Bullock et al., 2021).

Education and interventions have demonstrated efficacy in improving sleep and psychological wellbeing in sports settings (Tuomilehto et al., 2017; Donohue et al., 2018), yet athletes fail to report these issues to support staff. Juliff et al. (2014) showed that over half of athletes do not seek help for sleep problems, whilst 13% are given sleeping pills with no actual diagnosis. The knowledge and attitudes toward both sleep and mental health within sport is poor. Further, only 43% of coaches and support staff surveyed by Miles et al. (2019) promoted sleep hygiene. For many coaches, dealing with athletes’ mental health issues is not considered part of their role (Lebrun et al., 2020). The consequence of coaches ignoring these issues not only perpetuates these negative stigmas, but inadvertently encourages unhealthy coping mechanisms. The unhealthy attitudes and negative stigmas preventing help seeking are enduring and can continue to prevent treatment following retirement (Gulliver et al., 2014). Some research, however, indicates that perceptions toward mental health are shifting in a positive direction. For example, 68% of coaches from one survey had spoken to athletes about mental health during the last season (Murphy and Sullivan, 2021). Athletes seem to want help, but they either do not know how or where to get it from, or fail to do so in fear of the perceived consequences associated with help seeking (e.g., negative evaluation, deselection or being removed from the team).

### Scope of Current Research

The majority of research surrounding sleep and mental health in sport has previously focussed on competing athletes, with little focussed on retired athletes. Post-retirement research is saturated by an emphasis on comorbidities, such as concussion history and subsequent chronic traumatic encephalopathy (Kerr et al., 2012, 2014b; Didehbani et al., 2013; Hart et al., 2013; Strain et al., 2013; Iverson, 2014; Hutchison et al., 2018; Hind et al., 2021; Schaffert et al., 2021), and obesity (Hyman et al., 2012; Churchill et al., 2018). Further, this research is often limited to contact sport athletes, recruiting male dominated samples. Therefore, research into different sports, female athletes, and different levels of competition receive little attention—particularly following retirement. Consequently, we cannot completely understand the extent to which mental health and sleep problems affect former athletes, nor do we know whether they are more susceptible to these problems than those currently competing across.

Research into current and former athletes has failed to compare the two cohorts. Weigand et al. (2013) found in United States collegiate athletes, depression levels were higher in current (16.77%) compared to former athletes (8.03%). However, college sport is not representative of most athletic populations, as athletes transition out of sport with a degree, equipping them with skills and resources to gain employment. By contrast, for professional athletes, elite sport is a full-time occupation and focus for much of their life; thus, career prospects outside of sport can seem limited (Eggleston et al., 2020). Evident through the literature presented, a large proportion of research on these two issues—particularly mental health—focuses on college athletes. As a result, the generalisability of these findings is limited. A recent meta-analysis by Gouttebarge et al. (2019) suggested that the prevalence of distress, sleep disturbance and anxiety/depression is higher in current athletes than in former athletes. However, the studies analysed did not explicitly compare cohorts and employed inconsistent screening tools–a recurring theme throughout the literature.

The methodologies used to screen for mental health issues in athletes lack consistency (Rao and Hong, 2016). Further, Tahtinen et al. (2021) identified 28 different measures used across studies to assess depression in athletes. For example, Weigand et al. (2013) employed the Wakefield Depression Scale, despite Kearns et al. (1982) demonstrating its poor ability to screen for depression and suggesting that it should be abandoned in research. Despite measuring the same construct, the cut-off scores used to diagnose depression differ. This is a recurring theme amongst sleep disorders screening tools too. A number of inconsistencies exist in the measures used to assess symptomology and diagnose sleep disorders—typically lacking specificity for athletic populations (Samuels et al., 2016; Driller et al., 2018). Without consistent screening tools we cannot accurately determine the prevalence and severity of these disorders amongst current and former athletes.

Qualitative interviews are underutilised in this field, despite their value in identifying and understanding problems during athletic retirement (Beamon, 2012; Brownrigg et al., 2012; López and Levy, 2013). Further, every athlete interviewed by Beamon (2012) described difficulty while transitioning out of their athletic role when retiring; 70% did not know who they were, or how others would relate to them without sport, and/or mentioned a loss of status associated with being an athlete. In addition, qualitative methods can identify ways that athletes themselves believe that sleep and mental health outcomes can be improved. Moreover, retired athletes interviewed by Brownrigg et al. (2012) suggested that players should be encouraged to pre-plan for career transitions. Future research would benefit by employing quantitative and qualitative methods simultaneously to measure sleep and mental health.

## Future Directions

Future research must determine the prevalence and severity of sleep and mental health disorders amongst current and retired athletes, to allow and encourage those with previously undiagnosed issues to seek help before they worsen. These actions may be necessary to safeguard athletes from the acute and chronic problems that poor sleep and mental ill-health pose. There is an evident need for consistency in the screening tools used to measure sleep and mental health issues in this field. As suggested by Gouttebarge et al. (2019), by using questionnaires designed for and validated in athletic populations, we could reliably screen for mental health symptoms and disorders in athletes. The same principle applies when assessing sleep disorders across current and former athletes (Samuels et al., 2016; Driller et al., 2018). For example, the Athlete Sleep Screening Questionnaire (Samuels et al., 2016; Bender et al., 2018), Centre for Epidemiologic Studies Depression Scale—Revised (Eaton et al., 2004; Poucher et al., 2021), and Generalised Anxiety Disorder—7 (Spitzer et al., 2006; Tran, 2020) are either validated in athletic populations or possess the necessary psychometric properties to do so. Combining these questionnaires would create a specific tool to screen for sleep complaints and mental health issues in current and retired athletes, whilst applicable to the general population to allow cohort-based comparisons. It is important that this research is conducted in a broader context than what has previously been accomplished. Obtaining data from a large cross section of competing and former athletes from different levels of competition, genders, and sports would allow generalisability of findings and significantly expand the narrow scope of current literature. Interviewing retired athletes would help us understand the issues predisposing and perpetuating factors relating to the transition out of sport. Athletes themselves can explain how retirement can be handled better, indicating the support required from coaches and sporting organisations to facilitate successful transitions. By combining these research methodologies, we could confidently evaluate the state of sleep and mental health during and after athletic careers.

Longitudinal sleep assessment in these populations would also allow comparisons between cohorts and enable the identification of disordered and poor sleep. With modern wearable technologies, such as activity monitors (Sargent et al., 2016) and nearable sleep monitoring devices (e.g., Withings Sleep Analyser Mat) (Edouard et al., 2021), conducting this research on a large-scale is feasible. Technological advancements in portable polysomnography (e.g., Dreem Headband) (Arnal et al., 2020) offers a non-invasive, at-home method of analysing the microarchitecture of sleep. Combining validated questionnaires with home-based sleep monitoring would have immense value for the early identification of potential sleep and psychological disorders. Thus, the influence of mental health on sleep can be assessed, which has yet to be undertaken in sport. Sleep behaviours of those in remission from depression return to normal, at both a macro and micro level (Wichniak et al., 2013). Results reinforce that it is never too late to seek treatment for these issues, which can subsequently improve performance, health, and wellbeing. Sport can come down to small margins, thus, improvements to psychological wellbeing and sleep can translate to improvements in on-field performance. For example, simply increasing sleep opportunities for basketball players led to quantifiable increases in athletic performance (i.e., sprint speed, shooting accuracy) (Mah et al., 2011). Future research can highlight the role of mental ill-health on sleep, and the value of help-seeking for mental health problems. As such, evidence-based education can facilitate successful transitions and help prevent the deleterious health effects associated with sleep and mental health issues.

## Conclusion

Competitive sport and the transition out of it adversely affects sleep and psychological wellbeing. The stigma surrounding these issues continue to deteriorate wellbeing and prevent treatment, culminating into clinical disorders. Yet, no study has comprehensively compared sleep and mental health disorders between current and former athletes from different populations and competition levels. Previous research has focussed on either current or former athletes (or has failed to explicitly compare the two), specific cohorts (i.e., student athletes, specific sports, specific genders), and fails to utilise consistent screening tools. Consequently, while we understand why these issues occur, we do not truly know how many athletes suffer from them. More research in this field is necessary to identify and treat sleep and mental health disorders both during and after sport, before they worsen.

Future research would benefit from employing large-scale surveys, longitudinal sleep measurement, and qualitative interviews across all codes and levels of sport. This research would enable us to identify the prevalence and severity of sleep and mental health disorders in these populations, and subsequently broaden the current scope of the literature. Improving the athletes’ and sporting organisations’ awareness of these issues will facilitate and encourage help-seeking behaviour to make sport and the transition into retirement easier and healthier. We know that sleep and mental health interventions are successful and can improve outcomes. While it would be beneficial to implement these strategies to all athletes, being able to identify those who have symptomology or meet criteria of sleep and mental health disorders should be the initial target audience. From which, treatment can prevent these issues manifesting (e.g., worsening into a clinical disorder, causing other comorbid issues, suicide, etc.). By undertaking research in a much broader context than which has already been achieved, qualitative and quantitative data can highlight just how common these issues are and potentially shift the negative stigma associated with help-seeking in sport.

## Author Contributions

AM was responsible for the conceptualization and primary production of this literature review. DS, RA, and MD supervised AM during this process and assisted in editing this manuscript for final production. All authors contributed to the article and approved the submitted version.

## Conflict of Interest

The authors declare that the research was conducted in the absence of any commercial or financial relationships that could be construed as a potential conflict of interest.

## Publisher’s Note

All claims expressed in this article are solely those of the authors and do not necessarily represent those of their affiliated organizations, or those of the publisher, the editors and the reviewers. Any product that may be evaluated in this article, or claim that may be made by its manufacturer, is not guaranteed or endorsed by the publisher.
